# High-dose ivermectin in malaria and other parasitic diseases: a new step in the development of a neglected drug

**DOI:** 10.1051/parasite/2018039

**Published:** 2018-07-16

**Authors:** Olivier Chosidow, Charlotte Bernigaud, Giao Do-Pham

**Affiliations:** 1 Department of Dermatology, Hôpital Henri-Mondor, AP–HP Créteil France; 2 Research group EpiDermE (Epidémiologie en Dermatologie et Evaluation des Thérapeutiques) EA 7379, Université Paris-Est Créteil Val-de-Marne Créteil France; 3 French Satellite of the Cochrane Skin Group Créteil France; 4 Research Group Dynamyc, EA7380, Ecole nationale vétérinaire d’Alfort, Maisons-Alfort, Université Paris-Est Créteil Créteil France; 5 Department of Internal Medicine, Centre Hospitalier Intercommunal de Créteil Créteil France

**Keywords:** Oral ivermectin, High doses, Malaria, Head lice, Scabies

## Abstract

We highlight the absence of high-level evidence from dose-ranging studies regarding the use of oral ivermectin in susceptible parasitic diseases. We provide published data supporting the use of a higher dosage regimen of ivermectin in malaria and difficult-to-treat head lice, and announce an ongoing randomized clinical trial in severe scabies.

## Abstract (fr)

Nous soulignons l’absence de données probantes de haut niveau sur les études de dosage concernant l’utilisation de l’ivermectine par voie orale dans les maladies parasitaires sensibles. Nous fournissons des données publiées soutenant l’utilisation d’un régime plus élevé d’ivermectine dans le paludisme et les poux de tête difficiles à traiter, et nous annonçons un essai clinique randomisé en cours dans la gale sévère.

In a recent issue of the Lancet Infectious Diseases Journal, Smit et al. [12] reported a randomized clinical trial showing the good efficacy/tolerability ratio of a high-dose regimen of ivermectin (i.e., 300 μg/kg per day for 3 days) in uncomplicated malaria. Indeed, oral ivermectin is one of the major weapons against various parasitic diseases such as onchocerciasis and helminthiasis, and in 2015, Omura and Campbell received the Nobel Prize in Medicine for their discovery. However, the clinical development of ivermectin lacks high-level dose-ranging studies, and the dose of 150–200 μg/kg was considered the standard regimen for years for millions of people presenting ivermectin-susceptible parasitic diseases (https://www.accessdata.fda.gov/drugsatfda_docs/label/2008/050742s022lbl.pdf [9]).

In the 1990s, clinical and parasitological resistance [2]_,_ followed by genetic resistance [8] of head lice to the neuro-toxic effects of pyrethroid insecticides was found, leading to the discovery of therapeutic alternatives. Since lice are blood-feeding parasites, oral ivermectin 200 μg/kg was conceptually an interesting option, but the efficacy was disappointing because a single dose eradicated head lice infestation in only 6 of 26 subjects (23%) in a non-controlled study.[6]

Unpublished in-house company data from Merck Sharpe & Dohme-Chibret led to the use of ivermectin 400 μg/kg, 7 days apart, which was compared to 0.5% malathion lotion insecticide in a household-cluster randomized clinical trial [4]. The superiority of the high-dose ivermectin regimen in patients with difficult-to-treat head lice was clearly demonstrated (95.2% of patients receiving ivermectin were lice-free on day 15, as compared with 85.0% of those receiving malathion: absolute difference, 10.2 percentage points; 95% confidence interval, 4.6–15.7; *p* < 0.001). The frequency of adverse events did not differ between the two treatment groups.

Cases of scabies reach around 100–130 million yearly, and the condition is distributed worldwide [1]. It represents a significant global burden [3, 7], involving school absenteeism, psycho-social impacts, and impetigo and its complications, in particular in low-resource countries. This explains why scabies has recently been added to the WHO’s list of neglected tropical diseases (http://www.who.int/neglected_diseases/diseases/en). Oral ivermectin and topical 5% permethrin are considered the drugs of choice in many countries (topical benzyl benzoate may be used too) [5]. In classical scabies (with a limited number of mites on the skin), although ivermectin at 200 μg/kg seems the drug of choice for mass administration [10], a recently published Cochrane systematic review did not detect a large difference with 5% topical permethrin at week 2 (with low-certainty evidence) [11]. From our previous findings in head lice, we hypothesize that a higher dosage of ivermectin could be more appropriate, especially in patients with highly parasitized severe scabies. Therefore, we are conducting a French Ministry of Health-approved randomized clinical trial (Programme Hospitalier de Recherche Clinique 2014 AOM14612) in severe scabies (i.e., profuse and crusted scabies, with dozens to millions of mites on the skin), comparing ivermectin 400 μg/kg–200 μg/kg, 3 doses, 7 days apart (in combination with 5% topical permethrin and emollients in both groups) (https://clinicaltrials.gov; NCT02841215).

We believe that a high-dose ivermectin regimen should be better investigated in parasitic diseases, as has been done in malaria and ectoparasitosis.

## Conflicts of interest

Olivier Chosidow: Research grant from Codexial, Speaker fees from Zambon and Codexial; Charlotte Bernigaud: Research grant from Codexial; Giao Do-Pham: none.
